# Impact of the articulating robotic system on suprapancreatic lymphadenectomy in obese patients with gastric cancer: A comparative study with conventional laparoscopy

**DOI:** 10.1007/s11701-026-03258-z

**Published:** 2026-03-09

**Authors:** Hajime Kashima, Shunya Hanzawa, Yoshihiko Kakiuchi, Satoru Kikuchi, Shinji Kuroda, Shunsuke Kagawa, Toshiyoshi Fujiwara

**Affiliations:** 1 Department of Gastroenterological Surgery, Okayama University Graduate School of Medicine, Dentistry and Pharmaceutical Sciences, Okayama, Japan; 2 2-5-1 Shikata-cho, Kita-ku, 700-8558 Okayama, Japan

**Keywords:** Gastric cancer, Obesity, Robotic gastrectomy, Lymph node dissection, Suprapancreatic area

## Abstract

Obesity complicates lymph node dissection (LND) during gastric cancer surgery, particularly in deep anatomical zones. While robotic gastrectomy (RG) offers ergonomic advantages, its impact on the technically demanding suprapancreatic area in obese patients remains unclear. This study compared LND quality between RG and laparoscopic gastrectomy (LG) in obese patients.We retrospectively analyzed 404 patients who underwent LG (n = 319) or RG (n = 85) for gastric cancer. Patients were stratified by body mass index (BMI) (non-obese < 25 kg/m^2^ vs. obese ≥ 25 kg/m^2^). We assessed retrieved lymph nodes in the suprapancreatic area (stations 7, 8a, 9) and perigastric area (stations 1–6). Two-way analysis of variance (ANOVA) assessed the effects of BMI and surgical approach.In the perigastric area, obesity was an independent risk factor for decreased lymph node yields (p = 0.033), with no significant difference between RG and LG (p = 0.50). Conversely, in the suprapancreatic area, the robotic approach was a strong independent factor for increased retrieval (p < 0.001). Pairwise comparisons showed that obesity significantly reduced yields in the LG group (total: p = 0.012; suprapancreatic: p = 0.015), while the RG group maintained consistently high yields regardless of BMI (p = 0.371 and p = 0.857, respectively).The robotic approach was associated with superior, more consistent suprapancreatic LND, whereas obesity negatively affected perigastric retrieval regardless of platform. These findings suggest that robotic articulation may help maintain the quality of suprapancreatic lymphadenectomy in obese patients.

**Supplementary Information:**

The online version contains supplementary material available at 10.1007/s11701-026-03258-z.

## Introduction

Gastric cancer remains a leading cause of cancer-related mortality worldwide, and minimally invasive surgery (MIS) has firmly established itself as the standard of care for its treatment. Large-scale randomized controlled trials, such as JCOG0912 [1] and KLASS-01 [2], have demonstrated that laparoscopic gastrectomy (LG) is non-inferior to open surgery in terms of oncological safety, while offering the benefits of reduced postoperative pain and faster recovery. However, the applicability of LG is not uniform across all patient populations. A significant challenge that persists in the field of gastrointestinal surgery is the management of obese patients [3, 4]. As the global prevalence of obesity rises, surgeons are increasingly encountering patients with high body mass index (BMI) who require complex oncological procedures.

Obesity is not merely a physical characteristic but a complex pathological condition that significantly impacts surgical risk. As highlighted by Pasulka et al., obesity is associated with severe impairments in cardiac and pulmonary reserve, which, combined with metabolic anomalies, elevate the risk of perioperative morbidity [5]. Furthermore, excessive visceral adipose tissue (VAT) characterizes a "hostile abdomen," physically restricting the working space and obscuring critical anatomical landmarks [6]. Ri et al. emphasized that obesity serves as a definitive surgical risk factor, correlating with increased rates of surgical site infections, anastomotic leakage, and prolonged operative time [4]. In the specific context of gastric cancer, these factors complicate lymph node dissection (LND), particularly around the suprapancreatic area, where major vessels are deeply embedded in dense, vascularized fat. Consequently, previous studies have indicated that obesity is an independent risk factor for retrieving fewer lymph nodes during LG, potentially compromising accurate staging and oncologic outcomes [7].

The robotic surgical system, with its articulating instruments and stable 3D visualization, potentially overcomes these limitations. Previous studies have demonstrated the safety and feasibility of robotic gastrectomy [8–10]. Advanced robotic techniques, such as the preemptive retropancreatic approach and full intracorporeal robotic-sewn anastomosis, have been developed to enhance the precision of gastric surgery [11, 12]. These advantages are particularly noted in total gastrectomy, where robotic assistance facilitates complex D2 lymphadenectomy [13]. However, whether robotic technology can specifically mitigate the technical consistency issues of lymphadenectomy in obese patients remains a subject of debate.

In this study, we focused on the differential impact of obesity on two anatomical zones: the perigastric area (anatomically simple) and the suprapancreatic area (anatomically complex). We aimed to determine whether the robotic approach provides a specific technical advantage in maintaining LND quality in obese patients, using a two-way ANOVA.

## Methods

### Study design and patient selection

This single-center retrospective cohort study reviewed 404 consecutive patients who underwent radical gastrectomy for primary gastric adenocarcinoma between January 2017 and December 2022. Inclusion criteria were: (1) histologically proven gastric adenocarcinoma; (2) curative resection (R0) with lymphadenectomy according to the Japanese Gastric Cancer Treatment Guidelines [14]; and (3) minimally invasive surgery (MIS) approach, either laparoscopic gastrectomy (LG) or robotic gastrectomy (RG). Exclusion criteria included open conversion, palliative resection, distant metastasis, and remnant gastric cancer. Neoadjuvant chemotherapy was not an exclusion criterion, and such patients were included in the primary analyses to reflect real-world practice. Patients were categorized into four groups based on the surgical approach (laparoscopic or robotic) and obesity status: Non-obese (BMI < 25 kg/m^2^) and Obese (BMI ≥ 25 kg/m^2^). This cutoff aligns with the World Health Organization (WHO) expert consultation for Asian populations [15] and the Japan Society for the Study of Obesity (JASSO) [16].

### Data collection

Clinical characteristics, operative data, and postoperative outcomes were retrospectively collected from electronic medical records. Baseline characteristics included age, sex, BMI, comorbidities (diabetes mellitus, cardiovascular disease), tumor location, pathological stage, and preoperative/postoperative chemotherapy status. Operative details, including the type of gastrectomy, extent of lymphadenectomy, operative time, and estimated blood loss, were recorded.

### Surgical approach selection

The choice of surgical approach (robotic or laparoscopic) was not based on specific patient-related clinical factors or tumor characteristics. Instead, patient assignment was primarily determined by institutional logistical factors, including the availability of the robotic surgical system and the scheduled surgical staff for each operating day. During the study period, robotic gastrectomy was gradually adopted at our institution, and the proportion of robotic cases among minimally invasive gastrectomies increased over time. Consequently, both groups can be considered to have been selected without intentional clinical bias.

### Surgical procedures

All surgeries were performed by board-certified gastroenterological surgeons experienced in both laparoscopic and robotic techniques. Omentum management was not platform-specific. Total omentectomy was not routinely performed; the extent of omentum resection was determined according to the Japanese Gastric Cancer Treatment Guidelines and intraoperative judgment based on tumor factors.

### Laparoscopic gastrectomy (LG)

Performed using a standard 5-port technique with non-articulating instruments. A 12-mm camera port was placed at the umbilicus. A 5-mm port was placed in the right subcostal area, and a 5-mm or 12-mm port was placed slightly caudal to the midline of the right abdomen. Similarly, a 5-mm port was placed in the left subcostal area, and a 5-mm or 12-mm port was placed slightly caudal to the midline of the left abdomen. Specimen extraction was performed via a 3–4 cm mini-laparotomy at the umbilicus. 

### Robotic gastrectomy (RG)

Performed using the da Vinci® Surgical System (Intuitive Surgical, Sunnyvale, CA, USA). The robotic platform was utilized to perform meticulous dissection along the pancreas and major vessels, leveraging articulated instruments and 3D high-definition visualization. The camera port was placed at the umbilicus via a 3–4 cm incision equipped with a wound retractor and cover; this served as the 2nd arm. An 8-mm robotic port (1st arm) was placed in the right subcostal area. A 12-mm assistant port was inserted slightly cephalad between the camera port and the 1st arm. An 8-mm robotic port (4th arm) was placed in the left subcostal area, and another 8-mm port (3rd arm) was placed slightly caudal between the camera port and the 4th arm. Operative time was defined as the interval from skin incision to completion of skin closure for both approaches; therefore, robot docking time was included in the robotic operative time.

### Outcomes measured

The primary outcome was the total number of retrieved lymph nodes and the number retrieved at each station. Secondary outcomes included operative time, estimated blood loss (EBL), postoperative complications categorized by the Clavien-Dindo classification [17] (grade ≥ III considered major complications), length of hospital stay, overall survival, and recurrence-free survival.

### Evaluation of lymph node dissection

We categorized regional lymph nodes into two groups based on their anatomical location. (1) Perigastric area: Stations 1, 2, 3, 4sa, 4sb, 4d, 5, and 6. (2) Suprapancreatic area: Stations 7, 8a, and 9. Station 11p was excluded from the suprapancreatic analysis to eliminate bias arising from the difference in the proportion of D2 lymphadenectomy between the groups.

### Statistical analysis

Continuous variables are reported as mean ± standard deviation (SD) or median (interquartile range), as appropriate. Categorical variables are presented as numbers and percentages. To evaluate the number of retrieved lymph nodes, a two-way analysis of variance (ANOVA) was used to assess the independent effects of surgical approach and obesity, as well as their interaction. For specific station-by-station comparisons, pairwise comparisons between non-obese and obese groups within each surgical approach were performed using Welch’s t-test with Holm correction. Perioperative outcomes were compared among the four groups using one-way ANOVA followed by the Tukey–Kramer test for normally distributed variables (e.g., operative time), and the Kruskal–Wallis test followed by the Steel–Dwass test for skewed variables (e.g., estimated blood loss and length of stay). Categorical variables, including postoperative complications and chemotherapy rates, were evaluated using the chi-square test or Fisher’s exact test for overall comparisons. To further analyze the influence of factors on categorical outcomes, logistic regression analysis was used to calculate p-values for main effects and interactions. Survival and recurrence outcomes were analyzed using the Kaplan–Meier method, and differences between groups were evaluated using the log-rank test. Statistical significance was defined as p < 0.05. All statistical analyses were performed using JMP® Pro 17 (SAS Institute Inc., Cary, NC, USA).

## Results

### Patient characteristics

**Fig. 1:**
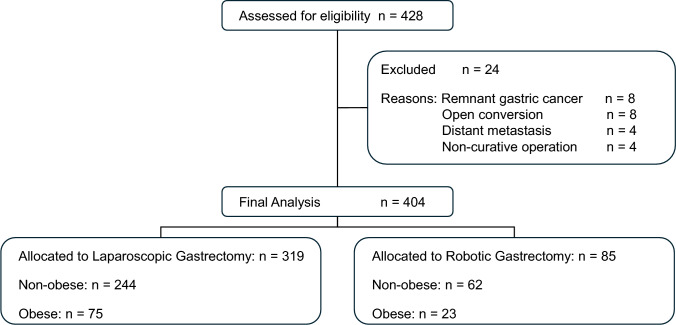
Study flowchart of patient inclusion and grouping**.** A total of 428 patients underwent minimally invasive gastrectomy during the study period. After excluding patients who did not meet the inclusion criteria, 404 patients were included in the final analysis. They were stratified into four groups based on the surgical approach (laparoscopic vs. robotic) and BMI category (non-obese < 25 kg/m^2^ vs. obese ≥ 25 kg/m^2^)

The baseline characteristics of the 404 eligible patients are summarized in Table 1. The total number of patients was 428, of whom 24 were excluded due to remnant gastric cancer, open conversion, or non-curative factors (distant metastasis or palliative resection) (Fig. 1). The prevalence of obesity was comparable between the laparoscopic (23.4%) and robotic (27.1%) groups. Regarding surgical background, the extent of lymphadenectomy differed significantly among groups (p = 0.005), with a higher proportion of lymphadenectomy greater than D2 in the Robotic Non-obese group (50.0%) than in the Laparoscopic groups (27.3%–28.0%). Other parameters such as age, sex, tumor location, comorbidities, and the administration of neoadjuvant (p = 0.705) and adjuvant chemotherapy (p = 0.858) were well-balanced across the four groups.

**Table 1:** Baseline characteristics of patients according to surgical approach and BMI category

Variables	Laparoscopic non-obese (n = 244)	Laparoscopic obese (n = 75)	Robotic non-obese (n = 62)	Robotic obese (n = 23)	Overall p-value†
Age, years	69.6 ± 10.8	68.7 ± 10.6	70.0 ± 11.3	67.5 ± 10.8	0.719
Male sex, n (%)	158 (64.8)	55 (73.3)	41 (66.1)	16 (69.6)	0.563
BMI, kg/m^2^	21.6 ± 2.0	27.8 ± 2.9	21.8 ± 2.3	27.5 ± 1.6	< 0.001
Comorbidities, n (%)					
Diabetes mellitus	31 (12.7)	17 (22.7)	7 (11.3)	4 (17.4)	0.147
Cardiovascular disease	28 (11.5)	8 (10.7)	9 (14.5)	2 (8.7)	0.86
Tumor location, n (%)					
Upper third	52 (21.5)	23 (30.7)	16 (25.8)	6 (26.1)	0.625
Middle third	84 (34.7)	27 (36.0)	22 (35.5)	9 (39.1)	
Lower third	106 (43.8)	25 (33.3)	23 (37.1)	8 (34.8)	
Pathological stage, n (%)					
I	167 (68.4)	52 (69.3)	38 (61.3)	15 (65.2)	0.872
II	43 (17.6)	15 (20.0)	16 (25.8)	4 (17.4)	
III	34 (13.9)	8 (10.7)	8 (12.9)	4 (17.4)	
Type of gastrectomy, n (%)					
Distal gastrectomy	183 (74.6)	50 (66.7)	43 (69.4)	14 (60.9)	0.082
Total gastrectomy	36 (14.7)	8 (10.7)	13 (21.0)	5 (21.7)	
Proximal gastrectomy	26 (10.7)	17 (22.7)	6 (9.7)	4 (17.4)	
Extent of lymphadenectomy, n (%)					
≦ D1 +	159 (65.2)	51 (68.0)	26 (41.9)	14 (60.1)	0.008
≧ D2	85 (34.8)	24 (32.0)	36 (58.1)	9 (39.9)	
Neo adjuvant chemotherapy, n (%)	27 (11.1)	7 (9.3)	5 (8.1)	1 (4.4)	0.705
Adjuvant chemotherapy, n (%)	43 (17.6)	12 (16.0)	13 (21.0)	5 (21.7)	0.858

Values are presented as mean ± standard deviation or number (percentage)

^†^P-values were calculated using one-way ANOVA for continuous variables and Pearson’s chi-square test or Fisher’s exact test for categorical variables

BMI, body mass index; D1 + , D1 plus lymphadenectomy; D2, D2 lymphadenectomy

### Perioperative outcomes

Table 2 details the perioperative outcomes and complications. The operative time was significantly longer in the robotic group compared to the laparoscopic group (Overall p < 0.001). Pairwise comparisons revealed that while obesity significantly prolonged operative time in the laparoscopic group (p = 0.041), no significant difference was observed between non-obese and obese patients in the robotic group (p = 0.834). Regarding estimated blood loss, the laparoscopic obese group had significantly higher blood loss compared to the non-obese group (median 100 vs. 10 mL, p < 0.001). In contrast, the robotic approach mitigated this increase, with the obese robotic group showing no significant difference compared to the non-obese robotic group (median 100 vs. 50 mL, p = 0.404). Regarding postoperative recovery, the Obese-Robot group had a shorter median hospital stay (11 days) than non-obese patients. In terms of safety, the incidence of any complications (p = 0.633) and major complications (Clavien-Dindo ≥ III, p = 0.174) did not differ significantly among the groups. Although the incidence of pancreatic fistula showed an overall significant difference (p = 0.042), pairwise analysis revealed no significant pairwise differences between specific groups. No cases of pancreatic fistula, anastomotic leakage, or intra-abdominal abscess were observed in the Obese-Robot cohort. Regarding short-term mortality and recurrence, no 30-day postoperative mortality was observed in the Lap Non-obese group (1 case, 0.4%), and there was no significant difference (p = 0.884).

**Table 2:** Perioperative outcomes and complications according to surgical approach and BMI category

Variables	Laparoscopic non-obese (n = 244)	Laparoscopic obese (n = 75)	Robotic non-obese (n = 62)	Robotic obese (n = 23)	Overall p-value^†^	Pairwise comparisons^‡^
Operative time, min	291.4 ± 86.6	323.2 ± 85.3	388.0 ± 94.2	406.3 ± 104.2	< 0.001	Lap Obese > Lap Non-obese (p = 0.041)
						Robo Non-obese > Lap Non-obese (p < 0.001)
						Robo Obese > Lap Non-obese (p < 0.001)
						Robo Non-obese > Lap Obese (p < 0.001)
						Robo Obese > Lap Obese (p < 0.001)
						Robo Obese vs Robo Non-obese (p = 0.834)^§^
Estimated blood loss, mL	10 (0–100)	100 (20–200)	25 (0–100)	100 (5–200)	< 0.001	Lap Obese > Lap Non-obese (p < 0.001)
						Lap Obese > Robo Non-obese (p = 0.006)
Length of hospital stay, days	11 (10–13)	10 (9–12)	11 (9–12)	11 (9–12)	0.147	
Postoperative complications, n (%)						
Any complication	58 (23.7)	13 (17.3)	16 (25.8)	5 (21.7)	0.633	
Pancreatic fistula	2 (0.8)	0 (0.0)	3 (4.8)	0 (0.0)	0.042	None^¶^
Anastomotic leakage	1 (0.4)	3 (4.0)	1 (1.6)	0 (0.0)	0.092	
Intra-abdominal abscess	1 (0.4)	2 (2.7)	1 (1.6)	0 (0.0)	0.323	
Anastomotic stenosis	3 (1.2)	0 (0.0)	1 (1.6)	1 (4.3)	0.417	
Postoperative bleeding	2 (0.8)	0 (0.0)	2 (3.2)	0 (0.0)	0.238	
Postoperative ileus	2 (0.8)	0 (0.0)	1 (1.6)	0 (0.0)	0.707	
Chylous ascites	3 (1.2)	1 (1.3)	0 (0.0)	1 (4.3)	0.456	
Pneumonia	5 (2.0)	1 (1.3)	1 (1.6)	0 (0.0)	0.892	
Other infections	9 (3.7)	4 (5.3)	4 (6.5)	0 (0.0)	0.532	
Clavien–Dindo grade ≧ III	13 (5.3)	4 (5.3)	8 (12.9)	2 (8.7)	0.174	
30-day mortality, n (%)	1 (0.4)	0 (0.0)	0 (0.0)	0 (0.0)	0.884	
Recurrence, n (%)	22 (9.0)	7 (9.3)	8 (12.9)	1 (4.4)	0.654	

Values are presented as mean ± standard deviation, median (interquartile range), or number (percentage)

^†^P-values were calculated using one-way ANOVA for continuous variables and Pearson’s chi-square test or Fisher’s exact test for categorical variables

^‡^Certain non-significant pairwise results are intentionally included to demonstrate the lack of significant impact of obesity within the robotic group

^§^The comparison between Robo Obese and Robo Non-obese is shown to document the absence of a statistically significant difference

^¶^Although the overall P-value indicated a significant difference, pairwise comparisons using Sidak’s multiple comparison test showed no significant differences between any two specific groups

### Oncological outcomes

**Fig. 2:**
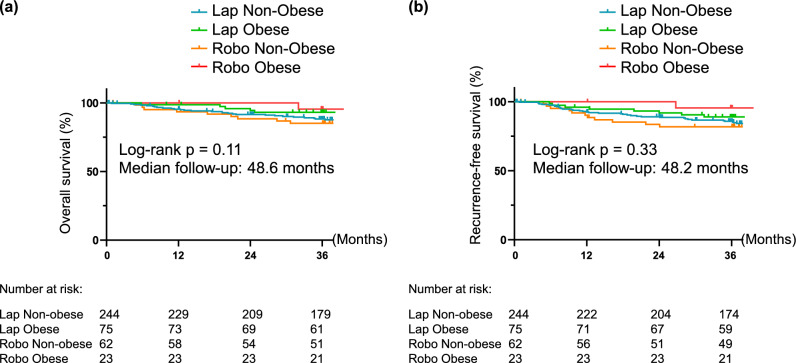
Kaplan–Meier survival curves for (**a)** overall survival and (**b)** relapse-free survival stratified by surgical approach and obesity. (**a)** Comparison of overall survival (OS) among the four subgroups. There were no significant differences in OS rates among the groups (p = 0.11). The 3-year OS rates were 88.3% (Lap/Non-obese), 93.1% (Lap/Obese), 84.8% (Robo/Non-obese), and 95.5% (Robo/Obese). (**b**) Comparison of recurrence-free survival (RFS) among the four subgroups. No significant differences were observed in RFS rates (p = 0.33). The 3-year RFS rates were 85.8% (Lap/Non-obese), 89.0% (Lap/Obese), 81.8% (Robo/Non-obese), and 95.5% (Robo/Obese)

**Fig. 3:**
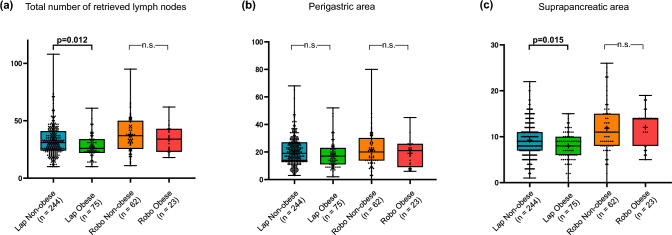
Comparison of retrieved lymph node counts stratified by surgical approach and obesity**.** Box-and-whisker plots representing the number of retrieved lymph nodes in the (**a)** total stations, (**b)** perigastric area (stations 1–6), and (**c)** suprapancreatic area (stations 7, 8a, and 9). Group sizes were: Lap/Non-obese (n = 244), Lap/Obese (n = 75), Robo/Non-obese (n = 62), and Robo/Obese (n = 23). (**A)** In the total station analysis, obesity significantly reduced yields in the laparoscopic group (p = 0.012), whereas the robotic group maintained consistent yields (p = 0.371). (**b)** In the perigastric area, no significant differences were observed between the surgical approaches or BMI categories. (**c)** In the suprapancreatic area, the robotic approach demonstrated a significant advantage over laparoscopy, including in obese patients (p < 0.001). While laparoscopic group showed a significant decline in yields due to obesity (p = 0.015), the robotic approach maintained high yields regardless of BMI (p = 0.857). Statistical significance was determined by Welch’s t-tests with Holm–Bonferroni correction following two-way ANOVA. *n.s.*, not significant

Kaplan–Meier survival analysis showed no significant differences in 3-year overall survival (OS) (Fig. 2a; p = 0.11) or 3-year recurrence-free survival (RFS) (Fig. 2b; p = 0.33) among the four groups. The 3-year OS rates were 88.3% in the Lap Non-obese group, 93.1% in the Lap Obese group, 84.8% in the Robo Non-obese group, and 95.5% in the Robo Obese group. Similarly, the 3-year RFS rates were 85.8% in the Lap Non-obese group, 89.0% in the Lap Obese group, 81.8% in the Robo Non-obese group, and 95.5% in the Robo Obese group. The median follow-up period was 48.6 months in OS and 48.2 months in RFS. These results suggest that robotic gastrectomy maintains both oncological safety and long-term survival, comparable to conventional laparoscopic outcomes, even in obese patients (Fig. 3).

### Lymph node retrieval

To evaluate the impact of surgical approach and obesity on lymphadenectomy quality, we performed a subgroup analysis of retrieved lymph nodes (Table 3) The mean total number of retrieved lymph nodes was significantly higher in the robotic group compared to the laparoscopic group (ANOVA main effect of approach, p < 0.001). Obesity was associated with a lower total lymph node count overall (main effect of BMI, p = 0.004). Crucially, the impact of obesity differed by approach. In the laparoscopic group, obese patients showed a significant reduction in total lymph node yield compared to non-obese patients (27.9 vs. 32.6, p = 0.012).

**Table 3:** Impact of surgical approach and obesity on lymph node retrieval: Two-way ANOVA results and subgroup analysis

Station		Number of harvested lymph nodes (mean ± SD)				2-way ANOVA P-value^†^			Pairwise comparisons P-value^‡^	
		Laparoscopic non-obese	Laparoscopic obese	Robotic non-obese	Robotic obese	Approach	Obesity	Interaction	Laparoscopic: non-obese vs obese	Robotic: non-obese vs obese
Total LNs		32.6 ± 13.8	27.9 ± 10.6	38.1 ± 15.7	33.9 ± 11.7	** < 0.001**	**0.004**	0.902	**0.012**	0.371
Perigastric Lymph nodes		20.9 ± 10.7	18.2 ± 10.1	21.9 ± 13.2	19.0 ± 9.9	0.5	**0.033**	0.96	0.293	1.00
	Station 1 (Right paracardial)	2.2 ± 2.3	1.9 ± 2.1	2.2 ± 2.1	2.3 ± 2.0	0.661	0.339	0.56	1.00	1.00
	Station 2 (Left paracardial)	1.9 ± 2.1	2.2 ± 1.5	2.3 ± 2.0	1.6 ± 1.1	0.841	0.97	0.268	1.00	1.00
	Station 3 (Lesser curvature)	6.5 ± 4.2	6.2 ± 4.6	6.9 ± 6.1	5.0 ± 3.1	0.999	0.188	0.209	1.00	0.38
	Station 4sa (Short gastric)	1.4 ± 2.4	0.6 ± 0.9	1.1 ± 1.2	0.9 ± 1.1	0.849	0.112	0.561	1.00	1.00
	Station 4sb (Left gastroepiploic)	1.1 ± 1.9	1.1 ± 1.9	1.4 ± 2.0	0.7 ± 1.1	0.69	0.369	0.222	1.00	0.34
	Station 4d (Right gastroepiploic)	6.2 ± 4.5	5.8 ± 4.5	6.2 ± 5.3	5.8 ± 4.8	0.999	0.53	0.967	0.44	1.00
	Station 5 (Suprapyloric)	0.8 ± 1.2	0.8 ± 1.0	0.8 ± 1.0	0.6 ± 0.9	0.448	0.58	0.639	1.00	1.00
	Station 6 (Infrapyloric)	4.5 ± 2.7	4.1 ± 2.7	4.9 ± 3.0	5.5 ± 2.9	0.1	0.6	0.181	0.14	1.00
Suprapancreatic Lymph nodes		9.2 ± 3.9	8.0 ± 3.3	11.8 ± 5.6	12.0 ± 4.2	** < 0.001**	**0.049**	0.19	**0.015**	0.857
	Station 7 (Left gastric)	3.0 ± 2.0	2.8 ± 2.1	4.2 ± 2.4	3.7 ± 2.5	** < 0.001**	0.269	0.643	0.777	0.777
	Station 8a (Common hepatic)	3.4 ± 2.0	3.2 ± 1.8	4.6 ± 3.0	4.6 ± 2.6	** < 0.001**	0.475	0.756	0.361	0.706
	Station 9 (Celiac)	2.9 ± 2.0	2.3 ± 1.8	3.5 ± 2.3	3.8 ± 2.1	** < 0.001**	0.131	0.155	**0.022**	0.499
	Station 11p (Proximal splenic)	2.0 ± 1.9	1.4 ± 1.5	2.1 ± 1.9	2.2 ± 2.2	0.338	0.204	0.301	0.795	0.795
	Station 11d (Distal splenic)	1.8 ± 1.8	2.3 ± 3.5	2.4 ± 1.9	1.2 ± 1.9	0.876	0.978	0.315	1.00	1.00
	Station 12a (Hepatoduodenal)	1.3 ± 1.1	1.8 ± 1.3	1.8 ± 1.5	1.7 ± 1.5	0.137	0.176	0.334	1.00	0.66

Values are presented as mean ± standard deviation

^†^Calculated by two-way ANOVA with surgical approach and obesity as factors

^‡^Pairwise comparisons between the non-obese and obese groups within each surgical approach were performed using Welch’s t-tests with Holm–Bonferroni correction

#### (1) Perigastric area (stations 1–6).

In the perigastric area, the two-way ANOVA revealed that obesity was an independent factor associated with decreased lymph node retrieval (p = 0.033), whereas the surgical approach had no significant impact (p = 0.50). No significant interaction was observed (p = 0.96). Both LG and RG groups showed a decreasing trend in yields with obesity (Lap/Non-obese: 20.9 vs. Lap/Obese: 18.2; Robo/Non-obese: 21.9 vs. Robo/Obese: 19.0).

#### (2) Suprapancreatic area (stations 7, 8a, 9)

In contrast, for the suprapancreatic area, the two-way ANOVA identified the robotic approach as a strong independent factor for increased retrieval (p < 0.001) and obesity as a significant independent factor for decreased retrieval (p = 0.049). However, pairwise comparisons with Holm–Bonferroni correction revealed that the negative impact of obesity was significant only in the LG group (Non-obese: 9.2 ± 3.9 vs. Obese: 8.0 ± 3.3, p = 0.015), whereas the RG group maintained consistently high yields regardless of BMI (Non-obese: 11.8 ± 5.6 vs. Obese: 12.0 ± 4.2, p = 0.857). To address potential confounding from differences in the extent of lymphadenectomy, we performed a sensitivity analysis restricted to D2 dissections. In this cohort, the robotic approach remained significantly associated with higher suprapancreatic lymph node retrieval (two-way ANOVA main effect of approach, p < 0.001), consistent with the primary analysis. (Details are provided in Supplementary Table S1.)

## Discussion

The present study demonstrated that the robotic approach provides superior lymph node retrieval in the suprapancreatic area compared to conventional laparoscopy. Previous reports in this journal have already established the safety and short-term benefits of robotic gastrectomy compared with laparoscopy [8, 9]. However, our study extends these findings by demonstrating that the specific articulating function of the robot effectively standardizes the quality of difficult suprapancreatic dissection, including in obese patients. The most significant finding is the contrast between the perigastric and suprapancreatic areas regarding the impact of obesity. Our two-way ANOVA revealed that the robotic approach was an independent factor enhancing lymph node yield. Most notably, while obesity significantly compromised the retrieval of suprapancreatic nodes in the laparoscopic group, the robotic group maintained a consistently high yield across BMI ranges. These findings strongly suggest that the robotic platform provides a “technical buffer” that neutralizes the anatomical challenges posed by visceral obesity. Because the extent of lymphadenectomy differed among groups, with a higher proportion of dissections beyond D2 in the Robotic Non-obese subgroup (Table 1) we performed a sensitivity analysis restricted to D2 dissections to minimize potential confounding by surgical intent or complexity (Table S1). In this D2-only cohort, the robotic approach remained significantly associated with greater suprapancreatic lymph node retrieval, consistent with the primary analysis and supporting the conclusion that the observed advantage is not solely attributable to differences in lymphadenectomy extent. While some subgroup pairwise comparisons were attenuated due to reduced sample size, the direction and magnitude of the effect were preserved.

Obesity has long been recognized as a formidable obstacle to lymphadenectomy [18, 19]. As noted in classic literature by Pasulka et al., the physiological burden of obesity involves not only cardiovascular strain but also physical impediments to surgery [5]. In laparoscopic gastric surgery, the thickness of the abdominal wall acts as a fulcrum that magnifies the force required to manipulate instruments, reducing tactile feedback and precision [20, 21]. Furthermore, as described by Ri et al., the visceral fat in obese patients is often friable and bleeds easily, which can obscure the surgical field and lead to inadvertent injury to adjacent organs [4]. Our data confirms that in the conventional laparoscopic setting, these factors lead to a statistically significant reduction in lymph node yield at the suprapancreatic nodes, a critical area for staging.

The superiority of the robotic approach observed in our study can be attributed to specific technological features. Coratti et al. discussed the current status of robotic gastrectomy, highlighting that the stable camera platform and the third arm allow for fixed retraction of the liver and stomach, creating a stable surgical field even in obese patients [22]. The articulated EndoWrist® instruments allow surgeons to reach behind the celiac artery and dorsal to the splenic vessels with angles impossible to achieve with straight laparoscopic instruments. The articulating capability of robotic instruments is particularly beneficial in technically demanding scenarios, such as in patients with a history of prior abdominal surgery, where limited maneuverability often hinders laparoscopic performance [9]. This capability facilitates "non-touch" isolation of the pancreas, preventing compression trauma even when the organ is embedded in thick visceral fat. This is clinically crucial because it ensures oncological compliance without compromising safety. Indeed, our observation of zero pancreatic fistulas in the Obese-Robot group, despite aggressive dissection, supports the technical safety profile described in previous reports. Furthermore, robotic surgery may reduce surgical stress, as evidenced by more favorable postoperative inflammatory responses [23]. Our results regarding the quality of suprapancreatic dissection are consistent with latest meta-analyses suggesting that the robotic platform facilitates thorough D2 lymphadenectomy compared to the conventional laparoscopic approach [24].

Several studies have highlighted the ergonomic benefits of the robotic platform. Our detailed analysis adds a new perspective by stratifying the impact of obesity based on anatomical complexity. In the perigastric area, we found no difference between the two approaches; obesity negatively affected both. This suggests that in anatomically superficial areas, the “volume” of visceral fat physically hinders retraction and pathological search, a challenge that even robotic instrumentation cannot fully resolve.

Regarding oncological safety, our study found no significant difference in overall survival between the laparoscopic and robotic groups, regardless of obesity status. This aligns with the findings of Liao et al., whose meta-analysis of over 3,000 patients confirmed that robotic gastrectomy is comparable to laparoscopic gastrectomy in terms of long-term oncological outcomes [25]. Similarly, Uyama et al. demonstrated the clinical advantages of RG in a prospective single-arm study, emphasizing its safety in early-stage cancer [26]. Building on these preliminary findings, Suda et al. recently reported in a multi-institutional study that RG yielded excellent 3-year outcomes for stage I/II gastric cancer, with a significantly higher overall survival rate compared to LG (96.3% vs. 89.6%, p = 0.009) [27]. Although our cohort included advanced stages, the consistent survival rates across groups suggest that the enhanced lymphadenectomy provided by the robot contributes to accurate staging and locoregional control, effectively preventing stage migration phenomena.

While this study establishes the technical benefits of RG in obese patients, the field is moving toward higher-level evidence and exploring future directions for the standardization of robotic technology [28]. Makuuchi et al. have recently published the protocol for the JCOG1907 (MONA LISA study), a randomized controlled phase III trial investigating the superiority of robot-assisted gastrectomy over laparoscopic gastrectomy for stage T1-4aN0-3 tumors [29]. The results of such trials will be pivotal to determining whether the technical advantages we observed—specifically, the enhanced suprapancreatic dissection—translate into a statistically significant survival benefit in a prospective setting. Finally, the issue of cost cannot be ignored. Robotic surgery is inherently more expensive than laparoscopy due to equipment and maintenance costs [30]. However, Lu et al. conducted a prospective trial-based economic evaluation comparing robotic, laparoscopic, and open gastrectomy [31]. They suggested that while the initial costs are higher, the reduction in complications and length of stay could offset the total financial burden. In our study, the Obese-Robot group had a shorter hospital stay than non-obese patients. If the robotic approach can reliably prevent costly complications like pancreatic fistula and reduce the length of stay in high-risk obese patients, it may prove to be cost-effective from a societal perspective, justifying its use in this specific demographic despite the higher upfront expense.

Our study has limitations inherent to its single-center retrospective design, and residual confounding cannot be fully excluded. Although patients receiving neoadjuvant chemotherapy were included and the proportion was low and comparable between groups, neoadjuvant treatment may still influence lymph node yield through treatment-related fibrosis or alterations in nodal architecture. The sample size of the Obese-Robot group was relatively small, which may have limited statistical power to detect subtle interaction effects and to evaluate rare outcomes; therefore, the absence of pancreatic fistula in this subgroup should be interpreted as hypothesis-generating rather than definitive. In addition, obesity was defined using BMI based on Asian criteria (BMI ≥ 25 kg/m^2^), which differs from Western standards and may not fully capture visceral adiposity; visceral fat area was not uniformly available in this cohort. Finally, gastrectomy type may affect the distribution and yield of certain lymph node stations, and although the procedure types were broadly comparable, some residual confounding remains possible. Despite these limitations, the consistent direction of the findings across multiple suprapancreatic lymph node stations supports the robustness of the observed association between the robotic approach and improved suprapancreatic lymphadenectomy; nonetheless, prospective multicenter studies incorporating direct measures of visceral adiposity are warranted.

In conclusion, robotic gastrectomy was associated with higher suprapancreatic lymph node retrieval in this retrospective cohort, including obese patients, without an apparent increase in major morbidity. Given the retrospective design and limited subgroup sizes, these findings should be interpreted cautiously and require prospective validation.

## Supplementary Information

Below is the link to the electronic supplementary material.

Supplementary file1 (XLSX 11 kb)

## Data Availability

The datasets generated and/or analyzed during the current study are available from the corresponding author on reasonable request.
